# Comparison of [^18^F]flortaucipir and [^18^F]MK6240 for the detection of tau pathology in Alzheimer’s disease (HEAD): a multicentre, prospective, cross-sectional, within-participant study

**DOI:** 10.1016/S0140-6736(26)00417-4

**Published:** 2026-05-30

**Authors:** Guilherme Povala, Bruna Bellaver, Firoza Z Lussier, Livia Amaral, Guilherme Bauer-Negrini, Pamela C L Ferreira, Andreia Rocha, Emma Ruppert, Marina S Medeiros, Cécile Tissot, William J Jagust, Joseph C Masdeu, Juan Fortea, Dana L Tudorascu, David N Soleimani-Meigooni, Val Lowe, Hwamee Oh, Belen Pascual, Brian A Gordon, Pedro Rosa-Neto, Suzanne Baker, Tharick A Pascoal

**Affiliations:** Department of Psychiatry, University of Pittsburgh, Pittsburgh, PA, USA (G Povala PhD, B Bellaver PhD, F Z Lussier MS, L Amaral MS, G Bauer-Negrini PhD, P C L Ferreira PhD, A Rocha PhD, E Ruppert MD, M S Medeiros MD, D L Tudorascu PhD, T A Pascoal MD PhD); Lawrence Berkeley National Laboratory, Berkeley, CA, USA (C Tissot PhD, Prof W J Jagust MD, D N Soleimani-Meigooni MD, S Baker PhD); Department of Neurology, Houston Methodist Research Institute and Weill Cornell Medicine, Houston, TX, USA (Prof J C Masdeu MD PhD, B Pascual PhD); Sant Pau Memory Unit, IR SANT PAU, Hospital de la Santa Creu i Sant Pau, Barcelona, Spain (J Fortea MD PhD); Barcelona Down Medical Center, Fundació Catalana Síndrome de Down, Barcelona, Spain (J Fortea); Center of Biomedical Investigation Network for Neurodegenerative Diseases (CIBERNED), Madrid, Spain (J Fortea); Edward and Pearl Fein Memory and Aging Center, University of California San Francisco, San Francisco, CA, USA (D N Soleimani-Meigooni); Department of Radiology, Mayo Clinic, Rochester, MN, USA (Prof V Lowe MD); Brown University, Department of Psychiatry and Human Behavior, Providence, RI, USA (H Oh PhD); Department of Radiology, Washington University in St Louis, St Louis, MO, USA (B A Gordon PhD); Translational Neuroimaging Laboratory, McGill University Research Centre for Studies in Aging, Douglas Research Institute, Montréal, QC, Canada (Prof P Rosa-Neto MD PhD); Peter O’Donnell Jr Brain Institute, University of Texas Southwestern Medical Centre, Dallas, TX, USA (Prof P Rosa-Neto); Department of Neurology, University of Pittsburgh, Pittsburgh, PA, USA (T A Pascoal)

## Abstract

**Background:**

Tau PET imaging has emerged as a critical biomarker for Alzheimer’s disease, informing diagnosis, staging, and therapeutic selection. We investigated whether PET tracer selection alters tau detection.

**Methods:**

We conducted a prospective, multicentre, non-randomised, within-participant comparison of [^18^F]flortaucipir (Tauvid), currently used in clinical settings in the USA and Europe, and [^18^F]MK6240, an investigational tau PET tracer. Participants were recruited from eight north American sites and underwent tau PET, amyloid-β (Aβ) PET, and detailed cognitive assessments. Tau PET with both agents was acquired within a 45-day window. Coprimary outcomes were the discriminative accuracy for Alzheimer’s disease-related cognitive impairment and the frequency of tau positivity in early medial temporal lobe (MTL) and late neocortical regions. The study is registered with ClinicalTrials.gov, NCT05361382.

**Findings:**

Between March 2, 2022, and Aug 27, 2025, 775 individuals were enrolled, with 682 completing all procedures (373 [55%] female, 309 [45%] male; 38 [6%] aged 19–27 years, 214 [31%] aged 50–65 years, and 430 [63%] aged 65–89 years). 32 (5%) participants identified as Hispanic or Latino. 637 (93%) identified as White, 24 (4%) as Black or African American, 16 (2%) as Asian, and five (1%) as other. In addition, 49 (7%) individuals were identified as being from a rural area. [^18^F]MK6240 showed greater accuracy than [^18^F]flortaucipir in distinguishing Alzheimer’s disease from non-Alzheimer’s disease impairment (area under the curve 0·93, 95% CI 0·89–0·95 *vs* 0·86, 0·75–0·91; p<0·0001). Among the older adults, tau positivity status was concordant in 560 (87%) for MTL and 603 (94%) for neocortical regions. In cognitively unimpaired participants, [^18^F]MK6240 identified twice as many MTL-positive cases as [^18^F]flortaucipir (n=54 [15%] *vs* n=23 [6%]). Prevalence ratio in Aβ-positive was 2·43 (95% CI 1·50–3·94; p=0·0003), identifying 23 additional cases per 100. Among discordant cases, 75 (89%) were [^18^F]MK6240-positive only and had higher Aβ burden (p<0·0001), APOEε4 frequency (p<0·0001), and cognitive impairment (p=0·0043) than those negative on both tracers. Neocortical tau positivity was more frequent with [^18^F]MK6240 than with [^18^F]flortaucipir in cognitively impaired individuals (80 [28%] *vs* 46 [16%]). Prevalence ratio in Aβ-positive was 1·74 (95% CI 1·32–2·29; p<0·0001), identifying 15 additional mild cognitive impairment and 21 dementia cases per 100.

**Interpretation:**

Tau PET tracer selection influences the frequency of detection of tau pathology across the ageing and Alzheimer’s disease spectrum. Compared with [^18^F]flortaucipir, [^18^F]MK6240 identified more individuals with tau pathology in cognitively unimpaired and cognitively impaired individuals, with direct implications for patient stratification in clinical trials and more precise guidance for therapeutic decision-making.

**Funding:**

National Institute on Aging.

## Introduction

Alzheimer’s disease is biologically characterised by the accumulation of amyloid-β (Aβ) plaques and tau neurofibrillary tangles.^1^ Aβ deposition begins early, while tau pathology more closely tracks cognitive decline.^1–3^ Aβ deposition alone might not result in imminent cognitive decline; in contrast, combined Aβ and tau PET abnormalities strongly predict clinical progression.^2,3^ Tau PET follows a stereotyped pattern of deposition across the Alzheimer’s disease continuum, beginning in the medial temporal lobe (MTL) and spreading throughout the neocortex, closely mirroring post-mortem Braak staging and enabling in vivo disease staging.^4,5^ Therefore, tau PET not only relates to Alzheimer’s disease-related cognitive impairment, but also provides biologically grounded staging.^6^ These characteristics position tau PET as a critical biomarker tool for diagnosis, staging, and patient selection in disease-modifying therapies and clinical trials.^7,8^

[^18^F]Flortaucipir (Tauvid) and [^18^F]MK6240 are the most widely used PET radioligands for imaging tau in Alzheimer’s disease. [^18^F]Flortaucipir use is based on detecting neocortical tau pathology, with reported limitations for detecting early tau deposition in the MTL.^9^ [^18^F] MK6240, developed subsequently, provides a higher signal-to-noise ratio and has been widely adopted in Alzheimer’s disease clinical trials.^10,11^ Whether the choice of tracer alters the detection of tau pathology and the staging of Alzheimer’s disease remains to be assessed. We therefore conducted a large, prospective, multicentre, within-participant, head-to-head comparison of [^18^F]flortaucipir and [^18^F]MK6240 to test the impact of tracer selection on Alzheimer’s disease detection and staging, with the goal of informing future use in clinical trials and practice.

## Methods

### Study design and participants

The HEAD study is a multicentre, prospective, cross-sectional study, conducted across eight sites in the USA and Canada. The cohort included young, healthy controls (aged 19–27 years) and older individuals (aged 50–89 years) who were cognitively unimpaired, had mild cognitive impairment (MCI), or had dementia. Full inclusion and exclusion criteria are provided in the appendix (p 4). Written informed consent was obtained from all participants. For participants unable to provide informed consent, proxy consent was obtained from a legally authorised representative. The study protocol was approved by the institutional review board of each site. Advarra served as the institutional review board of record for sites in the USA (protocol identification number Pro00061365). The study is registered with ClinicalTrials.gov, NCT05361382.

### Procedures

Upon enrolment, verification of eligibility was performed through confirmation of inclusion and exclusion criteria. All participants underwent standardised clinical and cognitive assessments using the National Alzheimer’s Coordinating Center Uniform Data Set, version 3.0^12^ or 4.0, containing Clinical Dementia Rating and a comprehensive neuropsychological examination including the Montreal Cognitive Assessment. The Mini-Mental State Examination was additionally administered to all participants. Diagnostic criteria are detailed in the appendix (p 4). Composite scores for five cognitive domains (memory, language, attention, executive function, and visuospatial ability) were computed following a validated protocol (appendix p 3).^13^

MRI scans were acquired on 3T scanners using harmonised protocols aligned with ADNI4 guidelines (appendix p 6).^14^ All participants underwent PET scanning with an Aβ tracer ([^1^^1^C]PiB, [^18^F]florbetaben, or [^18^F]NAV4694). Participants also underwent both [^18^F]flortaucipir and [^18^F]MK6240 tau PET within a 45-day interval (mean 33 days [SD 44]; appendix p 6). Paired T1-weighted and PET volumes were spatially normalised to the ICBM stereotaxic space (appendix p 4). Centiloid values were calculated and validated as previously established.^15^ The MTL region of interest (ROI) was composed by the entorhinal cortex and amygdala.^2^ The neocortex ROI was composed by regions comprising Braak V and VI. Tau positivity was defined for each tracer and ROI, with thresholds for positivity set at the mean plus 2·5 SDs of young, healthy adults.^2^ Young individuals were used exclusively for threshold determination. Sensitivity analyses were performed with alternative thresholds derived from different reference populations, including cognitively unimpaired middle-aged (50–65 years) Aβ-negative adults and the entire cognitively unimpaired older Aβ-negative cohort (50–87 years). For each reference population, thresholds were calculated at 2, 2·25, 2·5, 2·75, and 3 SDs from the mean (appendix p 10). Following quantitative classification, images underwent visual inspection to identify potential false positives in individuals with discrepant positivity status (appendix p 7). We also performed a sensitivity analysis using quantitative criteria only for tau positivity. Adapted Braak-like PET staging was used as previously described.^4^ For a detailed description of ROIs, see the appendix (p 7). If individuals did not follow Braak stages in a hierarchical fashion, where later stages can only be achieved if the individual is positive for the previous stages, they were considered Braak stage discordant.

### Statistical analysis

Group differences across tau positivity categories were tested using ANOVA with false discovery rate correction for multiple pairwise comparisons. Individuals were considered Aβ PET positive based on visual reads or if they had Centiloid values higher than 20. Sensitivity analyses were conducted using Aβ positivity defined by quantitative criteria alone or by visual reads alone. MCI and dementia groups were analysed together as cognitively impaired when the primary question was distinguishing Alzheimer’s disease-related from non-Alzheimer’s disease cognitive impairment. Sensitivity analysis with groups separated by diagnosis was also performed. Individuals were classified as MCI due to Alzheimer’s disease or Alzheimer’s disease dementia when Aβ PET was positive. Diagnostic accuracy was evaluated using receiver operating characteristic analyses (area under the curve [AUC] with 95% CI), with AUCs compared between tracers using DeLong tests. For voxelwise AUC analyses, tracer-specific AUC values were extracted within predefined regions and distributions were compared using Wilcoxon rank-sum tests. Prevalence ratios were estimated with Poisson regression and robust (sandwich) variance, comparing the relative likelihood of regional tau positivity for [^18^F]MK6240 with [^18^F]flortaucipir, while adjusting for Centiloid. The number of participants needed to identify one tau-positive individual was defined as 1/prevalence for each tracer. The number needed to detect (NND) was calculated as the reciprocal of the absolute difference in positivity rates between [^18^F]MK6240-only and [^18^F]flortaucipir-only cases. CIs were derived using 1000 bootstrap iterations. Logistic regression estimated tau and cognitive-domain positivity probabilities for [^18^F]flortaucipir and [^18^F]MK6240 as a function of Centiloid. Analyses were performed in R (version 4.4.0), VoxelStats (version 1.2) on MATLAB (R2024b Update 6), and Python. For the full statistical analysis description, see the appendix (p 8).

### Role of the funding source

The funder of the study had no role in study design, data collection, data analysis, data interpretation, or writing of the report.

## Results

775 individuals were enrolled to undergo paired tau PET scans with [^18^F]flortaucipir and [^18^F]MK6240 between March 2, 2022, and Aug 27, 2025, across eight sites in the USA and Canada. Of these, 70 individuals withdrew before completing study procedures, and 22 were unable to complete Aβ or tau PET and/or MRI due to technical limitations or other reasons, leaving 683 individuals. Following processing and quality control of imaging data, one individual was excluded due to anatomical abnormalities that precluded image processing, resulting in 682 participants for final analysis (appendix p 12). This included 38 (6%) young adults (aged 19–27 years) and 644 (94%) older adults (214 [31%] aged 50–65 years; 430 [63%] aged 65–89 years). Among the older adults, 362 (53%) were cognitively unimpaired (aged 50–87 years; 21% Aβ-positive), 183 (27%) were individuals living with MCI (aged 53–89 years; 59% Aβ-positive), and 99 (14%) had dementia (aged 50–88 years; 88% Aβ-positive; table). 373 (55%) participants were women and 309 (45%) were men (table). 32 (5%) participants identified as Hispanic or Latino. 637 (93%) identified as White, 24 (4%) as Black or African American, 16 (2%) as Asian, and five (1%) as other. In addition, 49 (7%) individuals were identified as being from a rural area.

Figure 1A shows average standardised uptake value ratio (SUVR) maps across diagnostic and biomarker-defined groups. [^18^F]Flortaucipir showed visually higher uptake across the cortex in all Aβ-negative individuals. [^18^F]Flortaucipir presented increased uptake in most of the cerebral cortex in cognitively unimpaired Aβ-positive individuals, whereas [^18^F]MK6240 exhibited higher uptake in the medial temporal cortex in these individuals. Both tracers presented increased SUVR values as clinical symptoms progressed in Aβ-positive individuals (figure 1A). Voxelwise AUC analyses confirmed that the MTL provided the strongest discrimination between MCI due to Alzheimer’s disease and non-Alzheimer’s disease cognitive impairment ([^18^F]flortaucipir 0·81 [95% CI 0·70–0·87], [^18^F]MK6240 0·87 [0·82–0·92]; p<0·0001; figure 1B; appendix p 13). For differentiating Alzheimer’s disease dementia from non-Alzheimer’s disease cognitive impairment, in addition to the MTL ([^18^F]flortaucipir 0·86 [0·75–0·91], [^18^F]MK6240 0·93 [0·89–0·95]; p<0·0001), the lateral temporal ([^18^F]flortaucipir 0·87 [0·74–0·94], [^18^F]MK6240 0·90 [0·80–0·94]; p<0·0001) and precuneus ([^18^F]flortaucipir 0·81 [0·67–0·87]; [^18^F]MK6240 0·86 [0·75–0·90]; p<0·0001) also showed high discriminative performance (figure 1B; appendix p 13). ROI-based analyses yielded concordant results (appendix p 14).

In cognitively unimpaired participants, MTL tau positivity was detected in 23 (6%) with [^18^F]flortaucipir and 54 (15%) with [^18^F]MK6240. Stratified by Aβ status, nine (3%) and 20 (7%) cognitively unimpaired Aβ-negative individuals, with [^18^F]flortaucipir and [^18^F] MK6240 respectively, displayed MTL tau positivity, corresponding to a prevalence ratio of 2·22 (95% CI 1·06–4·64; p=0·0335). 14 (16%) and 34 (39%) cognitively unimpaired Aβ-positive individuals, with [^18^F]flortaucipir and [^18^F]MK6240 respectively, displayed MTL tau positivity, corresponding to a prevalence ratio of 2·43 (1·50–3·94; p=0·0003; figure 2A; appendix p 10). To identify one MTL tau-positive case, 6·3 cognitively unimpaired Aβ-positive individuals would need to be scanned with [^18^F]flortaucipir and 2·6 with [^18^F]MK6240 (figure 2C). [^18^F]MK6240 therefore identified an additional 23 cognitively unimpaired Aβ-positive individuals per 100 as tau-positive, compared with [^18^F]flortaucipir (NND 4·4, 95% CI 3·14–6·77; figure 2C). For neocortical tau PET positivity in cognitively unimpaired participants, only two individuals were positive for [^18^F]flortaucipir and five for [^18^F]MK6240. Among cognitively impaired participants, positivity rates for MTL tau were higher overall, with [^18^F]flortaucipir positive in 149 (53%) compared with 184 (65%) for [^18^F]MK6240. Stratification by Aβ status showed a significantly higher prevalence ratio for [^18^F]MK6240 only in cognitively impaired Aβ-positive individuals (1·19 [95% CI 1·09–1·31]; p=0·0002; figure 2B; appendix p 10). [^18^F]MK6240 identified an additional 16 cases with MCI and 12 with Alzheimer’s disease dementia per 100 as MTL tau-positive compared with [^18^F]flortaucipir (MCI Aβ-positive NND 6·3 [95% CI 4·32–12], Alzheimer’s disease dementia NND 8·5 [5·38–17·20]; figure 2C). For neocortical tau PET deposition, positivity was identified in 46 (16%) with [^18^F]flortaucipir compared with 80 (28%) with [^18^F]MK6240 (91% concordance among cognitively impaired, 94% whole population). Stratifying by Aβ status, only cognitively impaired Aβ-positive individuals showed a significantly higher prevalence ratio for [^18^F]MK6240 (1·74, 95% CI 1·32–2·29; p<0·0001; figure 2B). Prevalence ratio by clinical diagnosis is presented in the appendix (p 15). To detect one neocortical tau-positive case, 10·8 and 4·2 individuals with Aβ-positive MCI, and 2·5 and 1·6 individuals with Alzheimer’s disease dementia would need to be scanned with [^18^F]flortaucipir and [^18^F]MK6240, respectively (figure 2C). [^18^F]MK6240 identified an additional 15 MCI and 21 Alzheimer’s disease dementia cases per 100 as neocortical tau-positive (MCI Aβ-positive NND 6·7 [95% CI 4·5–12], Alzheimer’s disease dementia NND 4·8 [3·31–7·81]; figure 2C). NND was not significant for cognitively impaired Aβ-negative individuals for MTL or neocortical tau PET deposition.

[^18^F]MK6240 reached 50% positivity probability at a lower Aβ burden than [^18^F]flortaucipir for both MTL and neocortical regions (figure 3A; appendix pp 16–18). The estimated Centiloid value for MTL tau PET positivity was 81 (95% CI 73–88) for [^18^F]flortaucipir and 53 (46–59) for [^18^F]MK6240 (figure 3A, B). At 108 Centiloids (96–121), [^18^F]MK6240 reached 90% probability of MTL tau positivity, whereas [^18^F]flortaucipir reached the same threshold at 143 Centiloids (129–158; appendix p 10). Both tracers reached MTL tau positivity before the 50% threshold for memory impairment (121 Centiloids, 108–133; appendix p 10). The corresponding window for neocortical tau PET positivity was 171 Centiloids (149–192) for [^18^F]flortaucipir and 129 Centiloids (118–140) for [^18^F]MK6240 (figure 3B). For neocortical tau PET deposition, [^18^F]MK6240 reached the threshold for positivity slightly after the threshold for memory impairment (129 Centiloids *vs* 121 Centiloids, respectively) and before the 50% threshold for impairment in executive function (132 Centiloids, 114–151), attention (186 Centiloids, 152–220), language (192 Centiloids, 154–230), and visuospatial function (274 Centiloids, 180–368; figure 3B). Neocortical [^18^F]flortaucipir reached the positivity threshold after the impairment thresholds for both memory and executive function (figure 3B).

Considering tau PET for early detection, MTL tau PET positivity was observed in nine (1%) individuals who were positive for [^18^F]flortaucipir only and 75 (12%) who were positive with [^18^F]MK6240 only, with the remaining 560 (87%) showing concordant results (appendix p 19). Bland-Altman analysis revealed significant proportional bias between tracers (appendix p 19). Among the 84 discordant cases, 75 (89%) were [^18^F]MK6240-only positive. Among [^18^F]flortaucipir-only positive individuals, five were cognitively unimpaired Aβ-negative and four cognitively impaired (three Aβ-negative). Among [^18^F]MK6240-only positive individuals, 36 were cognitively unimpaired (20 Aβ-positive) and 39 cognitively impaired (28 Aβ-positive; appendix pp 20–21). Average brain maps revealed that individuals with [^18^F]flortaucipir-only and [^18^F]MK6240-only positivity had elevated tracer uptake confined to the entorhinal cortex. Although [^18^F]MK6240 uptake was visually apparent in the entorhinal cortex among [^18^F]flortaucipir-only cases, it did not exceed the quantitative threshold for [^18^F]MK6240 positivity (figure 4A). Compared with individuals who were negative on both tracers, [^18^F]MK6240-only participants had higher prevalence of Aβ positivity (64% *vs* 17%; p<0·0001), higher Centiloid burden (mean 50·9 [SD 48·7] *v*s 10·3 [25·7]; p<0·0001), greater APOEε4 carriership (55% *vs* 28%; p<0·0001), and lower memory score (mean −0·63 [SD 0·93] *vs* −0·24 [SD 0·84]; p=0·0043). [^18^F]Flortaucipir-only individuals did not significantly differ from those individuals negative on both tracers (figure 4B–E). Sensitivity analyses showed that the relative difference in tracer positivity was similar across different methods of cutoff determination, as well as using only numerical categorisation for tau positivity (appendix pp 22–23).

For tau PET staging, both tracers broadly recapitulated Braak progression, with 32 (5%) individuals with [^18^F]flortaucipir and 30 (5%) with [^18^F]MK6240 not following a Braak-stage like pattern of tau deposition. However, [^18^F]MK6240 consistently identified a higher proportion of positive cases across all Braak stage regions compared with [^18^F]flortaucipir (appendix p 24). Individual-level concordance between [^18^F]flortaucipir and [^18^F]MK6240 was only moderate, with 394 (61%) participants assigned to the same Braak stages (figure 5A). Among discordant cases, 185 (95%) were staged higher by [^18^F]MK6240 and nine (5%) by [^18^F]flortaucipir. Among Braak-positive individuals, [^18^F]MK6240 assigned individuals one stage higher than [^18^F]flortaucipir (median 1, mean 1·08, 95% CI 0·94–1·21; figure 5B; appendix p 25).

## Discussion

Tau PET tracers enable in vivo assessment of tau tangle pathology, with important applications in diagnosis, prognosis, disease staging, and biomarker-based selection and monitoring of participants in clinical practice and therapeutic trials. [^18^F]Flortaucipir is currently the only US Food and Drug Administration-approved tau PET tracer for estimating the density and distribution of tau tangle pathology in adults with cognitive impairment being evaluated for Alzheimer’s disease, whereas [^18^F]MK6240 remains limited to research and clinical trial settings. [^18^F]MK6240 showed higher accuracy in distinguishing cognitive impairment due to Alzheimer’s disease from non-Alzheimer’s disease dementia. Compared with [^18^F]flortaucipir, [^18^F]MK6240 detected twice as many cognitively unimpaired Aβ-positive individuals with early MTL tau PET deposition and 1·74-times more cognitively impaired Aβ-positive individuals with neocortical tau pathology. [^18^F]MK6240 also identified tau deposition at lower levels of Aβ burden, and classified individuals at more advanced stages of tau pathology than [^18^F]flortaucipir.

Both tracers differentiated individuals with cognitive impairment related to Alzheimer’s disease from non-Alzheimer’s disease causes, with [^18^F]MK6240 showing superior diagnostic accuracy compared with [^18^F]flortaucipir across brain regions and clinical groups. These findings align with post-mortem evidence showing strong tracer binding in Alzheimer’s disease and minimal uptake in non-Alzheimer’s disease tauopathies.^16,17^ Our results reinforce earlier observations that tau PET signal closely correlates with Alzheimer’s disease-related cognitive impairment.^2,18^ [^18^F]MK6240’s enhanced performance likely reflects a mix of greater sensitivity to early tau deposition and reduced background signal. In contrast, [^18^F]flortaucipir showed higher and variable cortical signal in Aβ-negative individuals, particularly as symptoms progressed, complicating early stage differentiation. This variability, attributed to off-target binding, has been well documented.^19–21^ These differences are most clinically relevant in early disease stages, such as MCI, when tau pathology is emerging, diagnostic certainty is limited, and treatment decisions are time sensitive. Tracers with greater accuracy in this window might better identify individuals whose current symptoms reflect early Alzheimer’s disease pathology versus those without significant tau accumulation. Despite differences in binding profiles, both tracers showed similar patterns of diagnostic accuracy across regions and subgroups, supporting their shared target and their use in early diagnostic workflows and trial selection.

[^18^F]MK6240 identified tau positivity in the MTL at lower Aβ burden than [^18^F]flortaucipir. Both tracers became positive in the MTL before the onset of memory impairment, but [^18^F]MK6240 detected tau pathology at twice the rate of [^18^F]flortaucipir in cognitively unimpaired individuals. This discrepancy led to 12% of participants being classified as [^18^F]flortaucipir-negative but [^18^F]MK6240-positive, half of whom were cognitively unimpaired. These individuals were more likely to be Aβ-positive, had higher Centiloid values, and had greater APOEε4 carriership than those negative on both tracers, underscoring the pathological relevance of [^18^F]MK6240-only uptake. Such discordance is particularly relevant for preclinical trial design, where early detection of MTL tau pathology with PET is critical for participant enrolment. Phase 3 trials targeting preclinical Alzheimer’s disease populations are ongoing, and if successful, tau PET could be incorporated into therapeutic selection in clinical practice.^10^ [^18^F]MK6240-based selection would increase detection of early tau pathology in cognitively unimpaired Aβ-positive individuals, identifying 23 additional cases per 100 compared with [^18^F]flortaucipir. The sizeable subset of [^18^F]MK6240-positive only individuals who exhibited greater levels of Alzheimer’s disease-related pathology suggests that more sensitive tau PET tracers can identify individuals at the earliest stages of tau deposition, both in clinical trials and in routine practice. Importantly, [^18^F]MK6240 identified a higher proportion of tau PET positivity confined to the medial temporal cortex among cognitively unimpaired Aβ-negative individuals than [^18^F]flortaucipir (7% *vs* 3%, respectively), a pattern consistent with previous post-mortem reports of medial temporal neurofibrillary tangle pathology in Aβ-negative older adults.^22^ Nonetheless, future in vivo autopsy studies with closely timed tau PET and neuropathology could provide additional context for interpretation. An implication of these findings is that datasets acquired with different tau PET tracers should not be pooled without careful consideration. Tracer-specific binding characteristics, revealed only through rigorous within-participant assessments across the disease spectrum, introduce systematic differences in estimated tau burden and stage, thereby biasing combined analyses. These intrinsic differences also make harmonisation particularly challenging, especially in individuals with early tau deposition, in whom early signal might not be detected consistently across tracers. The large paired dataset generated by this study provides the foundation for next-generation harmonisation strategies by enabling direct modelling of relative binding profiles across tau PET tracers.

The approved clinical indication for tau PET imaging is the detection of advanced neurofibrillary tangle pathology extending into the neocortex.^23^ In the present study, [^18^F]MK6240 identified 1·74-times more tau-positive cases in this region compared with [^18^F]flortaucipir, detecting approximately 15 additional tau-positive cases in individuals with MCI and 21 additional tau-positive cases in individuals with Alzheimer’s disease dementia per 100 individuals. Neocortical [^18^F]MK6240 positivity emerged at Aβ burden levels similar to those observed at the onset of memory impairment, whereas [^18^F]flortaucipir reached its positivity threshold only after impairment in both memory and executive function. These findings have direct implications for the use of current anti-Aβ therapies. Although Aβ detection is necessary for the diagnosis of Alzheimer’s disease, there is broad consensus that tau PET abnormalities provide stronger evidence that cognitive impairment is attributable to Alzheimer’s disease rather than alternative causes. This view aligns with previous frameworks for preclinical Alzheimer’s disease,^24,25^ cognitive impairment due to Alzheimer’s disease,^6,26^ neuropathological diagnostic criteria,^27^ and expert opinions.^8^ The Donanemab trial required tau PET positivity for enrolment and showed greater clinical benefit among individuals with low to moderate stages of tau burden, highlighting the role of tau PET in treatment selection.^28^ The greater sensitivity and performance of [^18^F]MK6240 in identifying Alzheimer’s disease-related cognitive impairment enables detection of tau PET-positive cases at the earliest clinical stages, when individuals are most likely to benefit from disease-modifying therapies.

[^18^F]Flortaucipir and [^18^F]MK6240 both showed a Braak-like pattern of tau accumulation, but important differences emerged at the individual level. Despite broadly similar spatial distributions, discrepancies in stage assignments were observed, with over one-third of participants placed at different Braak stages depending on the tracer. [^18^F]MK6240 more often assigned individuals to higher stages, typically by one stage and occasionally by up to three stages relative to [^18^F]flortaucipir. Previous PET-to-autopsy studies indicated that [^18^F]flortaucipir lacks sensitivity for early stage tau pathology (Braak I–III), despite reliably detecting tau in more advanced stages, likely due to its variable binding affinity depending on tangle maturity.^9,16,17^ By contrast, [^18^F]MK6240 binds at high affinity to the core fibrils in mature tau tangles in a dose-dependent manner.^29^ These findings are consistent with the reported higher affinity of [^18^F]MK6240 for neurofibrillary tangles.^30,31^ However, [^18^F]MK6240 has not yet undergone comparable post-mortem validation. Future in vivo post-mortem validation would corroborate and extend the findings presented here. Clinically, [^18^F]MK6240’s tendency to assign individuals to later stages of Alzheimer’s disease progression has implications. Tau stage is closely linked to prognosis, and the choice of tracer can result in different stage classifications for the same individual. This variation might affect estimates of future cognitive decline, influence decisions about treatment eligibility, and inform prognostic counselling.

The strengths of this study include its large, well characterised, multicentre cohort, which enabled a robust within-individual, head-to-head comparison of the most widely used tau PET tracers. To the best of our knowledge, this represents the largest direct comparison of PET tracers to date. Standardised clinical and imaging protocols were applied across study sites, facilitating data integration. This design mitigates inter-site variability and enhances the robustness of findings. However, the limitations of the study should also be acknowledged. Although closely timed post-mortem correlation would be unrealistic at this scale, such data would strengthen conclusions. We applied established quantitative methods to define tau positivity and stage individuals for each tracer and performed sensitivity analyses with alternative thresholds. Nonetheless, future work could refine thresholds and ROIs to optimise concordance across tracers. Moreover, visual reads might capture subtle regional increases in uptake that can be obscured by background signal in quantitative analyses, warranting direct comparison of quantitative and visual approaches. Longitudinal data collection can clarify how tracer signals evolve over time and help determine the prognostic significance of differences in tracer-based classification. Despite deliberate efforts to enhance representativeness, yielding approximately 18% of participants from under-represented backgrounds, the cohort remained predominantly composed of White participants, which restricts generalisability.

In conclusion, [^18^F]flortaucipir and [^18^F]MK6240 yield similar topographical assessments of cortical tau pathology. However, [^18^F]MK6240 significantly detects tau accumulation at earlier stages, which is associated with abnormalities in other Alzheimer’s disease biomarkers. This earlier detection might refine Alzheimer’s disease staging and enhance the selection of individuals for therapeutic intervention in both clinical trials and routine practice.

## Supplementary Material

1

## Figures and Tables

**Figure 1:**
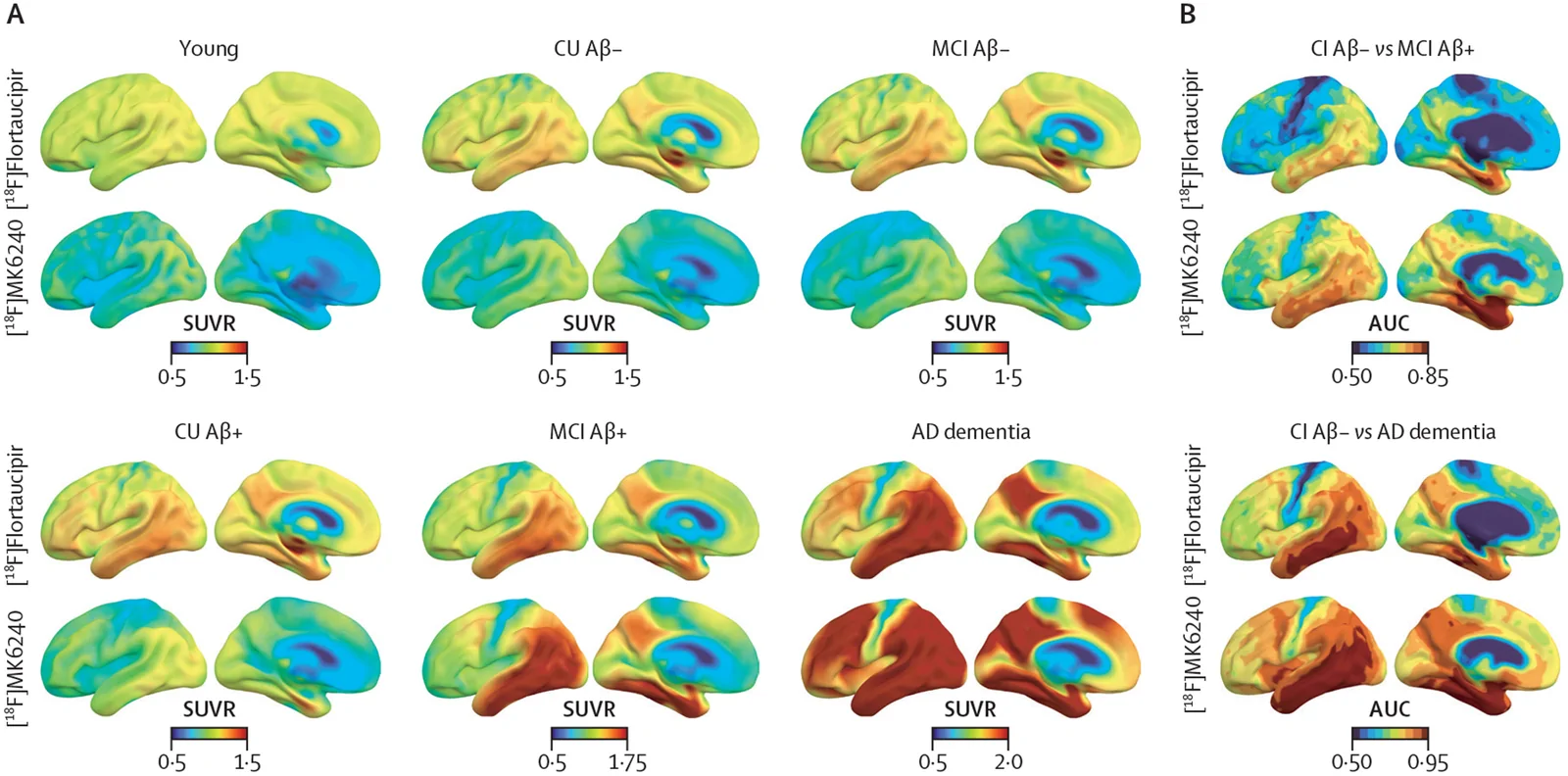
Discriminative accuracy of [^18^F]flortaucipir and [^18^F]MK6240 tau PET for distinguishing clinical groups (A) Voxelwise mean SUVRs for [^18^F]flortaucipir and [^18^F]MK6240 across different diagnostic and Aβ status groups. (B) Voxelwise AUC values for distinguishing MCI due to AD (top) or AD dementia from non-AD cognitive impairment disorders (bottom) using [^18^F]flortaucipir and [^18^F]MK6240. Participants with Alzheimer’s disease dementia were, by definition, Aβ-positive. Aβ–=amyloid-β negative. Aβ+=amyloid-β positive. AD=Alzheimer’s disease. AUC=area under the curve. CI=cognitively impaired. CU=cognitively unimpaired. MCI=mild cognitive impairment. SUVR=standardised uptake value ratio.

**Figure 2:**
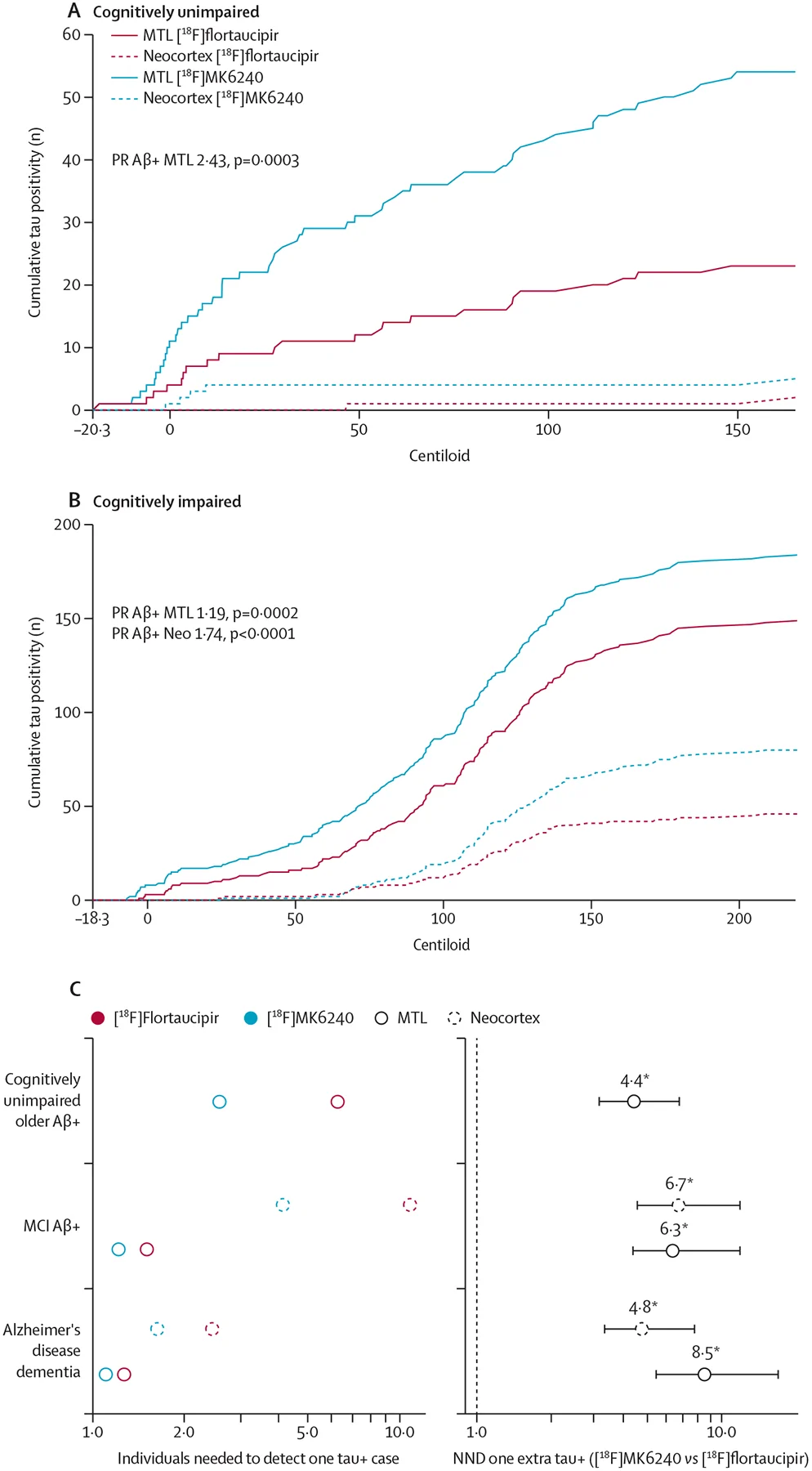
Frequency of [^18^F]flortaucipir and [^18^F]MK6240 tau positivity across cognitive status Cumulative tau MTL and neocortical positivity of [^18^F]flortaucipir and [^18^F]MK6240 as a function of Aβ burden (Centiloid) in cognitively unimpaired (A) and cognitively impaired (B) participants. (C) The number of individuals required to detect one tau-positive case and NND one extra tau positive case stratified by diagnostic and Aβ status. Neocortical region is composed of Braak V and VI regions. p values indicate whether detection of tau positivity differs between [^18^F]MK6240 and [^18^F]flortaucipir. Error bars indicate 95% CI. Asterisks indicate NND estimates for which the 95% CI of the paired absolute risk difference did not include zero. Participants with Alzheimer’s disease dementia were, by definition, Aβ-positive. Analysis accounted for Centiloid, age, sex, and study site. Aβ+=amyloid-β positive. PR=prevalence ratio. MCI=mild cognitive impairment. MTL=medial temporal lobe. Neo=neocortex. NND=number needed to detect. tau+=tau positive.

**Figure 3:**
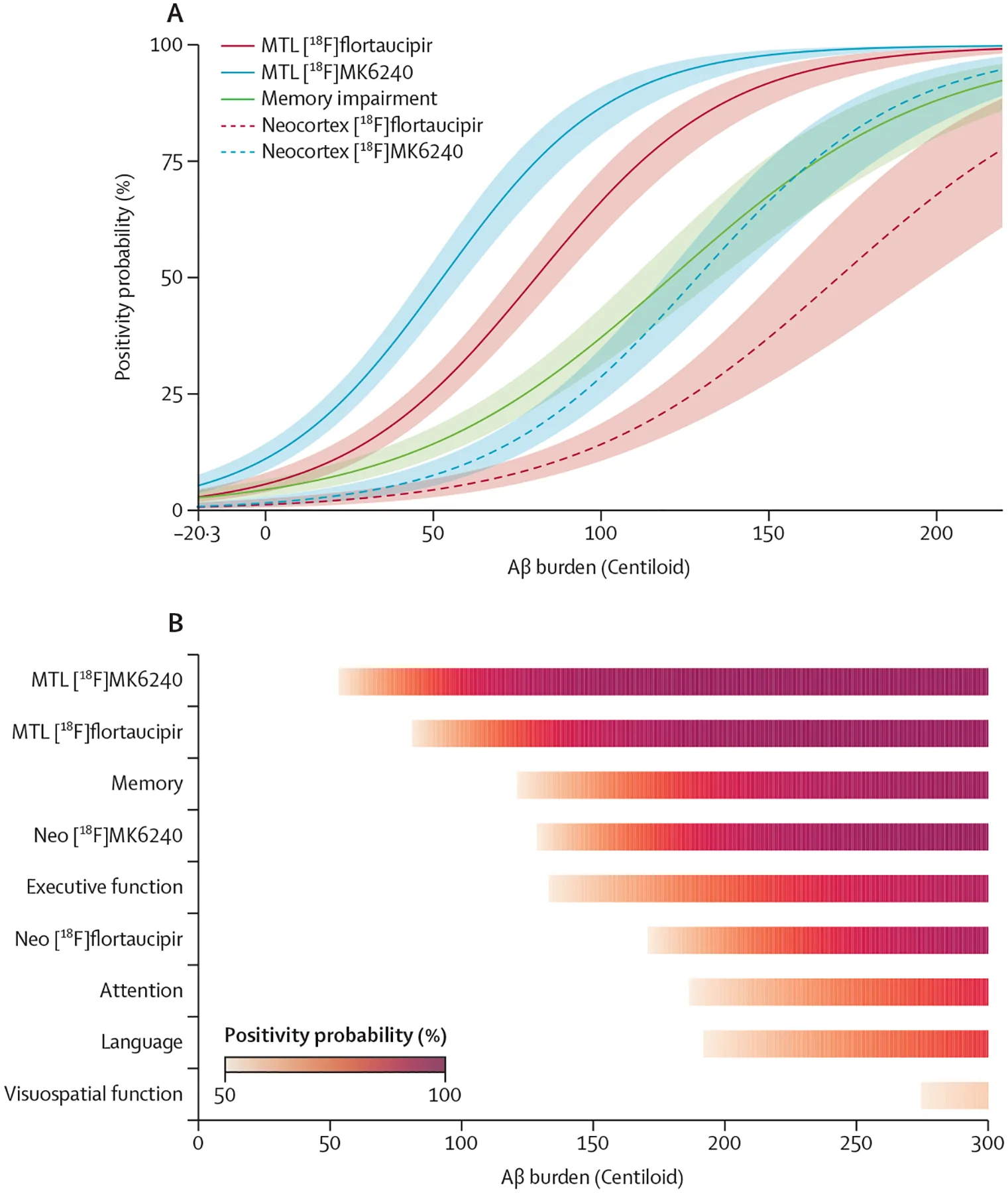
[^18^F]Flortaucipir and [^18^F]MK6240 tau positivity as a function of amyloid-β burden (A) Probability of tau positivity in the MTL and neocortex for [^18^F]flortaucipir and [^18^F]MK6240, and probability of memory impairment, as a function of Aβ burden (Centiloid). (B) Centiloid thresholds for 50% probability of tau positivity with each tracer and for 50% probability of decline in memory, executive function, attention, language, and visuospatial function. Shaded areas represent 95% CI. Aβ=amyloid-β. MTL=medial temporal lobe. Neo=neocortex.

**Figure 4:**
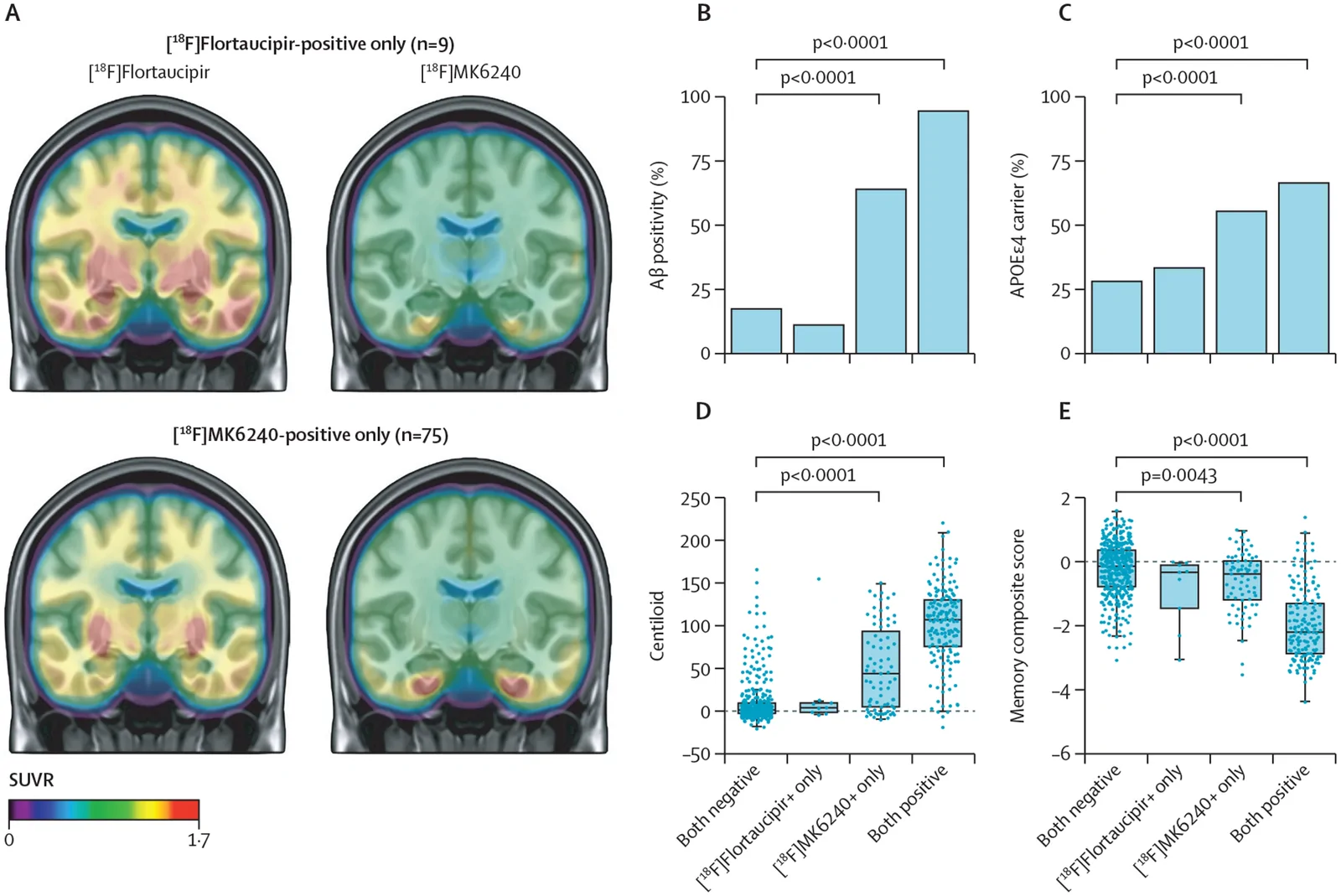
Discordant classifications of tau positivity with [^18^F]flortaucipir and [^18^F]MK6240 (A) Average SUVR maps of individuals positive only for [^18^F]flortaucipir (top) and only for [^18^F]MK6240 (bottom). Distribution of Aβ positivity (B), APOEε4 carriership (C), Centiloid levels (D), and memory composite score (E) according to [^18^F]flortaucipir and [^18^F]MK6240 status. Data are represented as median (IQR). Aβ=amyloid-β. Both negative=individuals negative on [^18^F]flortaucipir and [^18^F]MK6240. Both positive=individuals positive on [^18^F]flortaucipir and [^18^F]MK6240. SUVR=standardised uptake value ratio.

**Figure 5:**
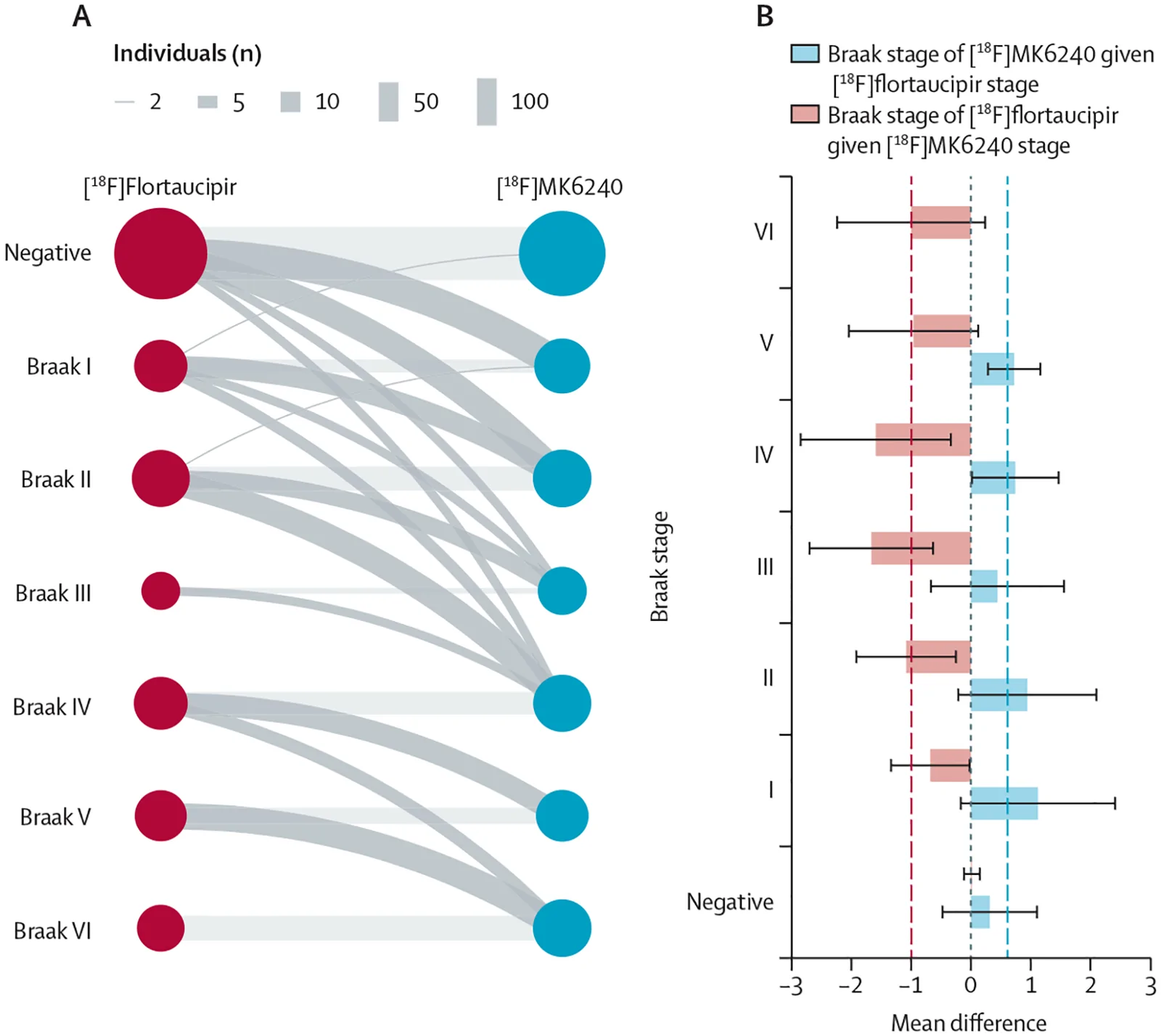
PET-based Braak staging using [^18^F]flortaucipir and [^18^F]MK6240 (A) Individual concordance in Braak stage classification between [^18^F]flortaucipir and [^18^F]MK6240. Node size reflects number of individuals per stage; links represent cross-classification between tracers. Links with only one individual were omitted. (B) Quantification of average Braak staging differences. The error bars represent 95% CI. The vertical dashed lines indicate the mean difference in Braak stage between tracers for each comparison direction. Individuals who did not follow Braak staging are not represented.

**Table:** Participant characteristics

	Young adults (n=38)	Older cognitively unimpaired adults (n=362)	Mild cognitive impairment (n=183)	Dementia (n=99)
Age	23 (2·0)	67 (8·3)	71 (7·4)	72 (8·9)
Sex				
Female	23 (61%)	220 (61%)	82 (45%)	48 (48%)
Male	15 (39%)	142 (39%)	101 (55%)	51 (52%)
*APOEε4* carrier	11 (29%)	109 (30%)	94 (51%)	59 (60%)
Education, years	16 (2·0)	16 (2·5)	16 (2·9)	15 (3·3)
MMSE score	29 (1·0)	29 (1·5)	28 (2·0)	21 (4·9)
CDR sum of boxes	0·0 (0·0)	0·02 (0·1)	1·7 (1·2)	4·7 (2·5)
Aβ positivity	0·0 (0·0%)	77 (21%)	108 (59%)	87 (88%)
MTL [^18^F]flortaucipir SUVR	1·0 (0·1)	1·1 (0·11)	1·3 (0·27)	1·5 (0·33)
MTL [^18^F]MK6240 SUVR	0·76 (0·1)	0·92 (0·24)	1·4 (0·70)	2·0 (0·82)
Neocortex [^18^F]flortaucipir SUVR	1·1 (0·09)	1·1 (0·08)	1·1 (0·14)	1·3 (0·35)
Neocortex [^18^F]MK6240 SUVR	0·89 (0·10)	0·91 (0·11)	1·0 (0·33)	1·6 (0·92)

Data are mean (SD) or n (%). Missing: five APOEε4 status, six education years, five MMSE score, and one CDR sum of boxes. Aβ=amyloid-β. CDR=Clinical Dementia Rating. MMSE=Mini-Mental State Examination. MTL=medial temporal lobe. SUVR=standardised uptake value ratio.

## Data Availability

Requests for raw or analysed data and materials should be directed to the corresponding author. All requests will be reviewed by the investigators and participating institutions to determine whether they are subject to intellectual property or confidentiality restrictions. Anonymised data will be shared with qualified academic investigators for the purpose of replicating the procedures and results reported in this Article. Data and materials will be made available under a material transfer agreement.

## Research in context

### Evidence before this study

We searched PubMed, Scopus, and Embase for studies published from database inception to Oct 20, 2025, using the terms “tau PET”, “T807”, “flortaucipir”, “Tauvid”, “MK6240”, “Alzheimer’s disease”, and “head-to-head tau PET” without language restrictions. Tau PET imaging has emerged as a critical biomarker for Alzheimer’s disease, informing diagnosis, staging, and therapeutic selection. [^18^F]Flortaucipir, the first introduced tau PET tracer, reliably detects advanced neocortical tau pathology, but has limited sensitivity for early tau deposition in medial temporal regions. [^18^F]MK6240, developed subsequently, potentially exhibits higher sensitivity for neurofibrillary tangles and superior signal-to-noise ratio. However, no previous large-scale study has compared these tracers within the same individuals across the Alzheimer’s disease spectrum, nor evaluated their relative performance for early detection, staging concordance, or diagnostic accuracy.

### Added value of this study

This multicentre, prospective, within-participant study represents, to the best of our knowledge, the largest head-to-head comparison of [^18^F]flortaucipir and [^18^F]MK6240 to date. Using harmonised acquisition protocols, standardised processing pipelines, and quantitative thresholds derived from young, healthy controls, we directly assessed the impact of tracer selection on tau detection, staging, and diagnostic discrimination. Compared with [^18^F]flortaucipir, [^18^F]MK6240 identified twice as many cognitively unimpaired Aβ-positive individuals with early medial temporal tau accumulation and more cognitively impaired Aβ-positive participants with neocortical tau involvement. [^18^F]MK6240 reached positivity at lower Aβ burden, detected tau pathology earlier in the disease continuum, and staged individuals on average one Braak stage higher. These findings show that tracer selection influences disease classification, staging, and eligibility for early intervention trials. They also prompt a re-evaluation of the natural history of tau PET positivity in vivo, which might have been underestimated due to limitations in tracer sensitivity.

### Implications of all the available evidence

Alzheimer’s disease affects over 55 million people worldwide, with rising prevalence and profound societal, economic, and health-care burden. Tau PET imaging, more closely aligned with symptom onset and disease progression than Aβ PET, is increasingly used to guide diagnosis, prognosis, and therapeutic enrolment. Our findings show that [^18^F]MK6240 enables earlier detection of tau pathology than [^18^F]flortaucipir, with direct implications for clinical trial design, biomarker qualification, and health system preparedness for early intervention strategies. These results suggest that current estimates of tau PET positivity might be substantially underestimated, potentially delaying recognition of tau pathology and limiting access to disease-modifying therapies informed by tau PET. Tracer selection should be considered a critical determinant in Alzheimer’s disease care pathways, regulatory frameworks, and global efforts to advance early detection, equitable access, and precision medicine in neurodegenerative disease.
