# Supplementary material for: Comparison of [18F]flortaucipir and [18F]MK6240 for the detection of tau pathology in Alzheimer’s disease (HEAD): a multicentre, prospective, cross-sectional, within-participant study
Source: Lancet. Author manuscript; Available in PMC 2026 Oct 12. (PMC13656316; doi:10.1016/S0140-6736(26)00417-4)
Supplement: Supplementary file 1 [file NIHMS2184177-supplement-1.docx]

**Supplementary Appendix**

Head-to-head comparison of tau PET tracers for Alzheimer’s disease detection and staging: a multicentre, prospective, cross-sectional study

Guilherme Povala^1^*, Bruna Bellaver^1^*, Firoza Z. Lussier^1^, Livia Amaral^1^, Guilherme Bauer-Negrini^1^, Pamela C.L. Ferreira^1^, Andreia Rocha^1^, Emma Ruppert^1^, Marina S. Medeiros^1^, William J. Jagust^2^, Joseph Masdeu^3^, Juan Fortea^4,5,6^, Dana L. Tudorascu^1^, David N. Soleimani-Meigooni^2,7^, Val Lowe^8^, Hwamee Oh^9^, Belen Pascual^3^, Brian A. Gordon^10^, Pedro Rosa-Neto^11,12^, Suzanne Baker^2^*, Tharick A. Pascoal^1,13^*^#^, the HEAD study.

*****These authors contributed equally to this work

^1^University of Pittsburgh, Department of Psychiatry, PA, United States of America.

^2^Lawrence Berkeley National Laboratory, Berkeley, CA, United States of America.

^3^Houston Methodist Research Institute, Department of Neurology, Houston, TX, United States of America.

^4^Sant Pau Memory Unit, IR SANT PAU, Hospital de la Santa Creu i Sant Pau, Barcelona, Spain.

^5^Barcelona Down Medical Center, Fundació Catalana Síndrome de Down, Barcelona, Spain.

^6^Center of Biomedical Investigation Network for Neurodegenerative Diseases (CIBERNED), Madrid, Spain.

^7^University of California San Francisco, Memory and Aging Center, San Francisco, CA, United States of America.

^8^Mayo Clinic, Department of Radiology, Rochester, MN, United States of America.

^9^Brown University, Department of Psychiatry and Human Behavior, Providence, RI, United States of America.

^10^Washington University in St. Louis, Department of Radiology, St. Louis, MO, United States of America.

^11^Translational Neuroimaging Laboratory, McGill University Research Centre for Studies in Aging, Douglas Research Institute, Montréal, QC, Canada.

^12^Peter O'Donnell Jr. Brain Institute (OBI), University of Texas Southwestern Medical Centre (UTSW), Dallas, USA

^13^University of Pittsburgh, Department of Neurology, PA, United States of America.

^#^Correspondence to:

Tharick A. Pascoal, MD, PhD

Associate Professor of Psychiatry and Neurology

Department of Psychiatry and Neurology, University of Pittsburgh, Pittsburgh, PA, USA

200 Meyran Avenue – Parkvale Building Room 506, Pittsburgh, PA, 15213, USA

E-mail: pascoalt@upmc.edu

**Table of Contents**

[Methods	4](#_Toc219477270)

[Participants	4](#_Toc219477271)

[Procedures	6](#_Toc219477272)

[Statistical analysis	8](#_Toc219477273)

[Supplemental Tables	10](#_Toc219477274)

[Supplemental Table 1. Mean, SD, and SUVR cut-offs by reference group.	10](#_Toc219477275)

[Supplemental Table 2. Sensitivity analyses of the frequency of tau PET positivity with Tauvid and MK6240 among Aβ-positive individuals, using alternative Aβ-positivity classification methods.	10](#_Toc219477276)

[Supplemental Table 3. Centiloid levels corresponding to different probabilities of tau or cognitive composite positivity.	11](#_Toc219477277)

[Supplemental Figure 1. Study flowchart.	12](#_Toc219477278)

[Supplemental Figure 2. Sensitivity analysis for the discriminative accuracy of Tauvid and MK6240 tau PET for distinguishing clinical groups.	13](#_Toc219477279)

[Supplemental Figure 3. Diagnostic performance of Tauvid and MK6240 for differentiating clinical groups.	14](#_Toc219477280)

[Supplemental Figure 4. Sensitivity analysis for the frequency of Tauvid and MK6240 tau positivity with the cognitively impaired group separated by diagnosis.	15](#_Toc219477281)

[Supplemental Figure 5. Average SUVR images for Tauvid and MK6240 across Centiloid (CL) ranges.	16](#_Toc219477282)

[Supplemental Figure 6. Tau tracer MTL and neocortical positivity stratified by Centiloid and clinical status.	17](#_Toc219477283)

[Supplemental Figure 7. Comparison of Tauvid and MK6240 tau positivity rates in the medial temporal lobe.	18](#_Toc219477284)

[Supplemental Figure 8. Cross-correlation and agreement between Tauvid and MK6240 in medial temporal lobe and neocortex.	19](#_Toc219477285)

[Supplemental Figure 9. Patterns of tau PET uptake in illustrative cases scanned with both Tauvid and MK6240.	20](#_Toc219477286)

[Supplemental Figure 10. Distribution of diagnostic groups across tau PET positivity patterns.	21](#_Toc219477287)

[Supplemental Figure 11. Sensitivity analyses using alternative MTL tau-positivity cut-off definition methods.	22](#_Toc219477288)

[Supplemental Figure 12. Sensitivity analysis of discordant classifications of tau positivity with Tauvid and MK6240 using quantitative criteria only.	23](#_Toc219477289)

[Supplemental Figure 13. Regional positivity frequencies across brain regions comprising Braak stages for Tauvid and MK6240 in Aβ+ participants.	24](#_Toc219477290)

[Supplemental Figure 14. Relationship between amyloid-β burden and probability of tau positivity across Braak stages for Tauvid and MK6240.	24](#_Toc219477291)

[Supplemental Figure 15. Concordance of Braak-like tau stages between Tauvid and MK6240.	25](#_Toc219477292)

[References	26](#_Toc219477293)

# Methods

## Participants

Study sample size was informed by estimated effect sizes for between-group differences in tau-PET across clinical groups, based on prior tau PET studies. Inclusion criteria were: (i) availability of a study partner able to provide an independent assessment of functioning; (ii) willingness and ability to undergo repeated MRI and PET imaging; and (iii) fluency in a language approved by the coordinating centre including English, Spanish, and French. Exclusion criteria were: (i) inability to provide informed consent (by self or by proxy); (ii) pregnancy or breastfeeding; or (iii) medical conditions or medications that, in the opinion of the investigator, increase risk to the participant or limit the ability to tolerate or interfere with study procedures or outcomes.

CU older adults were 50 years or older with a CDR of 0. Local physicians confirmed the presence of cognitive impairment.^1^ MCI was defined as subjective and objective evidence of cognitive decline, a CDR of 0.5, and largely preserved activities of daily living. Mild‑to‑moderate dementia met National Institute on Aging-Alzheimer’s Association criteria and had CDR scores between 0.5 and 2.

Our study sample included 18% of participants from underrepresented racial, ethnic, and rural groups. With respect to ethnicity, 4.5% of our sample reported as being Hispanic or Latino. With respect to race, 0.3% of our sample identified as being American Indian or Alaska Native, 2.8% identified as being Asian, and 3.8% identified as being Black or African American. In addition, 6.9% of our sample was identified as being from a rural area, identified using the Area Deprivation Index (ADI) from the University of Winsconsin-Madison’s Center for Health Disparities Research.^2^

Of the 775 individuals enrolled, 682 were included in the analyses. Of the 93 participants excluded, 70 withdrew prior to completing study procedures for personal reasons, 22 were unable to complete all imaging procedures due to technical limitations on the day of scanning or other reasons, and one participant was excluded during quality control due to anatomical abnormalities that precluded image processing. The 93 participants not included in the analyses had a mean age of 60 ± 20 years and were 53% female; 48% were cognitively unimpaired, 12% had mild cognitive impairment, 15% had dementia, and 20% were young healthy controls, with diagnostic information missing for 5%.

Minor protocol deviations were recorded at all study sites. Importantly, no protocol deviations in the HEAD study caused any harm or increased risk of harm to study participants, nor did they significantly adversely impact the integrity of our data. Recorded protocol deviations in the HEAD study fit into the following categories: study procedures conducted at a window greater than the protocol-required 45-day window; injected dose of a PET agent slightly below the target dose (most often by less than 0.5mCi); and missing MRI sequence(s) from the full MRI acquisition protocol. Recorded causes of the noted protocol deviations varied: for procedures conducted outside of protocol-required timelines, this was primarily due to study participant availability and scheduling constraints, but also due in some cases to PET scanner malfunction or maintenance requiring to reschedule a PET scan to the next available date; for injected PET tracer doses lower than the target dose, this was almost exclusively related to timing delays at the respective PET imaging center, with previous scans running late and injection times needing to be delayed; for missing MRI sequences, this was due to either technician error, MR scanner malfunction, or the study participant wanting to exit the MR scanner prior to the end of the full MR protocol. In all cases, study personnel at the respective study site mitigated the effects of the protocol deviations as much as possible, rescheduling study procedures to the soonest available dates, and ensuring that PET and MR protocols were properly implemented on all applicable scanners to avoid delays or missing acquisitions. The MR acquisition protocol used in the HEAD study is ordered such that the most important sequences are acquired first, minimizing any data loss. All protocol deviations are recorded in a central regulatory binder and reviewed by the PI of the study.

After initial approval of the HEAD study Master Protocol by a central IRB, minor changes were implemented, all of which were reviewed and approved by the IRB. Protocol modifications were composed of re-wording or existing language or added language in order to improve clarity and remove ambiguity in the description of study procedures. Shortly after enrollment into the study was initiated, an IRB-approved modification to the inclusion criteria for young healthy controls in the study expanded the age range for this group from 20-24 years to 18-28 years; this modification was implemented to facilitate the recruitment and enrollment of eligible young healthy controls into the study. No other important changes have been made to the HEAD study throughout its conduct.

## Procedures

Composite scores for five cognitive domains (memory, language, attention, executive function, and visuospatial ability) were computed with the mean of standardized z-scores derived from domain-specific neuropsychological tests, using a reference group of CU individuals with normal global cognition (CDR = 0, MoCA ≥ 27, MMSE ≥ 26)^3^. Composite scores for five cognitive domains were generated using the following tests: (i) The memory composite included immediate and delayed recall scores from the Craft Story 21 and the Benson Complex Figure Copy tests. (ii) The language domain incorporated scores from the Verbal Fluency (Phonemic), Category Fluency, and Multilingual Naming Test. (iii) Attention was measured using Trail Making Test Part A and Number Span Test. (iv) Executive function included Trail Making Test Part B and Number Span Test Backward. (v) Visuospatial ability was assessed using the Benson Complex Figure Copy immediate recall task.^3^ The vast majority of participants completed assessments in English, with 173 assessed in French and 9 in Spanish. Only 33 participants (5%) completed assessments in a language different from their mother language reducing the likelihood of systematic language-related bias. The first languages of these 33 participants included Arabic, Bengali, Greek, Hindi, and Hungarian, for which validated translations of the UDS were not available.

MRI scans were acquired on 3T scanners using harmonised protocols aligned with ADNI4 guidelines.^4^ High-resolution sagittal T1-weighted images were obtained using 3D accelerated MPRAGE or IRSPGR sequences. Intensity non-uniformities were corrected via N4 bias field correction, followed by segmentation into six tissue classes (grey matter, white matter, CSF, skull, soft tissue, and air) using DARTEL in SPM12. Whole-brain masks were generated by thresholding combined tissue probability maps of grey matter, white matter, and CSF (>90%) and filling internal voids. Cerebellar segmentation was performed with FreeSurfer v7.4.1 and FastSurfer v2.1.1 using the SUIT atlas. Native-space reference regions were defined as follows: the inferior cerebellar grey matter included bilateral Crus I/II, VIIb, VIIIa/b, IX, X, Vermis, and Vermis VII/VIII/IX. The whole cerebellum reference encompassed all SUIT-defined cerebellar areas. T1-weighted images were spatially normalised to the ICBM 2020 Nonlinear Symmetric template^5^ using ANTs (affine + SyN transformations). All resulting images and masks were visually inspected for accurate alignment.

All participants underwent PET scanning with Aβ and two tau PET tracers. Radiotracers were administered via intravenous bolus, following study-specific dosing protocols. Aβ PET imaging was conducted using one of three tracers: [¹¹C]PiB (~15 mCi), [¹⁸F]Florbetaben (~8 mCi), or [¹⁸F]NAV4694 (~8.1 mCi). Tau PET imaging involved head-to-head acquisitions with [¹⁸F]MK6240 (~5 mCi) and [¹⁸F]Tauvid (~10 mCi). PET images were reconstructed into four 5-minute frames using scanner-specific OSEM-based algorithms (e.g., VPHD, VPFX, TOF-OSEM) with standardised settings and no applied smoothing. Acquisition windows post-injection were: PiB (40-60 or 50-70 min), Florbetaben (90-110 min), NAV4694 (50-70 min), MK6240 (90-110 min), and Tauvid (80-100 min). All images were corrected for attenuation, motion, dead time, decay, scatter, and random events. PET scans were linearly co-registered to native T1-weighted MRIs using ANTs. Tau PET SUVRs were computed in native-space using the individual-specific mask of the inferior cerebellar grey matter. Aβ PET SUVRs were computed using the whole-cerebellum mask (as recommended for Centiloid calibration^6^). MRI-derived spatial transformations were applied to align PET images with the ICBM stereotaxic space, and whole-brain mask generated from each participant’s T1-weighted MRI was applied to exclude non-brain voxels. The final PET volumes were smoothed with a scanner-specific Gaussian kernel to achieve an 8 mm full width at half maximum (FWHM). Regions of interest (ROIs) were defined using the CerebrA atlas, which was modified to include a transentorhinal cortex segmentation generated using a validated protocol in 1-mm isotropic stereotaxic space.^7,8^ Regional SUVRs were extracted by computing the median SUVR within each ROI.^9^ Centiloid values were calculated using the standard composite ROI, which included the frontal, temporal, and parietal cortices, precuneus, anterior striatum, and insula.^6^ Adapted Braak-like PET staging was based on the following groupings: Braak I (transentorhinal cortex), II (entorhinal cortex and amygdala), III (parahippocampal, fusiform, and lingual gyri), IV (inferior and middle temporal cortices, caudal and rostral anterior cingulate, isthmus and posterior cingulate, and insula), V (frontal, parietal, and occipital cortices, transverse and superior temporal gyri, precuneus; pars opercularis, triangularis and orbitalis, supramarginal gyrus), and VI (precentral, postcentral, and paracentral gyri, cuneus; and pericalcarine cortex).^10^

Following quantitative classification of tau positivity, all images underwent visual inspection. Readers evaluated PET images overlaid on each participant’s MRI with ROI masks displayed. To maintain a conservative approach for tau-positivity classification and minimize potential false positives, individuals with discrepant positivity status between tracers were visually classified as negative when the signal was not clearly distinguishable from background or appeared to reflect spillover from adjacent regions. This determination was made by consensus among three readers who were blinded to the tracer used. Eighteen individuals (17 quantitatively positive for MK6240 and 1 quantitatively positive for Tauvid) were reclassified as negative after this visual review because the readers did not reach consensus on visual positivity.

## Statistical analysis

Diagnostic accuracy was assessed using ROC AUC. We generated whole-brain AUC maps by computing voxelwise classification performance for each tracer and group comparison. For each anatomical ROI, we extracted all voxel-level AUC values within that region and calculated the mean AUC across voxels as the regional summary statistic. Confidence intervals (95% CI) were derived from the 2.5th and 97.5th percentiles of the voxel-level AUC distribution within each region. Regional AUC distributions between tracers were compared using Wilcoxon rank-sum tests. In addition, we performed an ROI-based AUC analysis in which mean SUVR values were extracted from predefined anatomical regions for each subject, and standard ROC curves were generated for each region-of-interest and tracer. AUC values (95% CI) from this approach were compared between tracers using DeLong tests.

Prevalence ratios (PR) were estimated with Poisson regression and robust (sandwich) variance, comparing the relative likelihood of regional tau positivity for MK6240 vs. Tauvid while adjusting for continuous Aβ burden (Centiloid), age, sex, and cohort. The Poisson regression model was specified as: $log(E[Y]) = \beta₀ + \beta₁(Tracer) + \beta₂(Centiloid) + \beta₃(Age) + \beta₄(Sex) + \beta₅(Site)$, where $Y$ is the binary tau positivity outcome, $Tracer$ is an indicator variable for MK6240 (with Tauvid as reference), $Centiloid$ represents continuous Aβ burden, $Age$ is continuous, and $Sex$ and $Site$ are categorical covariates. The exponentiated coefficient $exp(\beta₁)$ provides the prevalence ratio for MK6240 relative to Tauvid, adjusted for all covariates. PRs were stratified by brain region (MTL, Neocortex) and Aβ status (Aβ+, Aβ−), with robust standard errors computed using the HC0 heteroscedasticity-consistent variance estimator.

The number needed to detect (NND) was calculated as the reciprocal of the absolute difference in positivity rates between MK6240-only and Tauvid-only cases, indicating how many individuals must be scanned with the more sensitive tracer instead of the less sensitive one to yield one additional case of tau PET positivity. Confidence intervals for the NND were derived using 1,000 bootstrap iterations, with statistical significance considered when the interval did not cross one.

**Results**

# Supplemental Tables

## Supplemental Table 1. Mean, SD, and SUVR cut-offs by reference group.

| **Region** | **Tracer** | **Mean** | **SD** | **Mean + 2SD** | **Mean + 2.25SD** | **Mean + 2.5SD** | **Mean + 2.75SD** | **Mean + 3SD** |
| --- | --- | --- | --- | --- | --- | --- | --- | --- |
| *Young controls (18-28 years old)* | | | | | | | | |
| MTL | Tauvid | 1.015 | 0.099 | 1.213 | 1.238 | 1.263 | 1.287 | 1.312 |
| MTL | MK6240 | 0.774 | 0.097 | 0.969 | 0.993 | 1.017 | 1.042 | 1.066 |
| Neocortex | Tauvid | 1.064 | 0.088 | 1.241 | 1.263 | 1.285 | 1.307 | 1.329 |
| Neocortex | MK6240 | 0.891 | 0.1 | 1.091 | 1.116 | 1.141 | 1.166 | 1.191 |
| *Middle-aged adults (50-65 years old, CU Aβ−)* | | | | | | | | |
| MTL | Tauvid | 1.064 | 0.088 | 1.241 | 1.263 | 1.285 | 1.307 | 1.329 |
| MTL | MK6240 | 0.845 | 0.1 | 1.044 | 1.069 | 1.094 | 1.119 | 1.144 |
| Neocortex | Tauvid | 1.051 | 0.077 | 1.205 | 1.224 | 1.243 | 1.262 | 1.282 |
| Neocortex | MK6240 | 0.916 | 0.096 | 1.109 | 1.133 | 1.157 | 1.181 | 1.205 |
| *CU elderly (50-87 years old, Aβ−)* | | | | | | | | |
| MTL | Tauvid | 1.087 | 0.1 | 1.286 | 1.311 | 1.336 | 1.36 | 1.385 |
| MTL | MK6240 | 0.877 | 0.133 | 1.142 | 1.175 | 1.209 | 1.242 | 1.275 |
| Neocortex | Tauvid | 1.044 | 0.076 | 1.196 | 1.215 | 1.234 | 1.253 | 1.272 |
| Neocortex | MK6240 | 0.908 | 0.102 | 1.113 | 1.139 | 1.164 | 1.19 | 1.215 |

Aβ: Amyloid-β; CU: cognitively unimpaired; MTL: medial temporal lobe; SD: standard deviation.

## Supplemental Table 2. Sensitivity analyses of the frequency of tau PET positivity with Tauvid and MK6240 among Aβ-positive individuals, using alternative Aβ-positivity classification methods.

| **Diagnosis** | **Region** | **PR** | **95% CI** | **P-value** |
| --- | --- | --- | --- | --- |
| *Centiloid > 20* | | | | |
| Cognitively impaired | MTL | 1.19 | [1.09 – 1.31] | 0.0001 |
| Cognitively impaired | Neocortex | 1.74 | [1.32 – 2.29] | 0.0001 |
| Cognitively unimpaired | MTL | 2.29 | [1.41 – 3.69] | 0.0007 |
| Cognitively unimpaired | Neocortex | 0.5 | [0.18 – 1.40] | 0.188 |
| *Visual Read* | | | | |
| Cognitively impaired | MTL | 1.19 | [1.09 – 1.31] | 0.0002 |
| Cognitively impaired | Neocortex | 1.74 | [1.32 – 2.29] | 0.0001 |
| Cognitively unimpaired | MTL | 2.43 | [1.49 – 3.97] | 0.0004 |
| Cognitively unimpaired | Neocortex | 0.5 | [0.18 – 1.40] | 0.188 |

PR adjusted for age, sex, and site. PR: prevalence ratio; CI: confidence interval; MTL: medial temporal lobe.

## Supplemental Table 3. Centiloid levels corresponding to different probabilities of tau or cognitive composite positivity.

| **Measure** | **25% prob**  **[95% CI]** | **50% prob**  **[95% CI]** | **75% prob**  **[95% CI]** | **90% prob**  **[95% CI]** |
| --- | --- | --- | --- | --- |
| MTL MK6240 | 25 [19, 31] | 53 [46, 59] | 81 [72, 89] | 108 [96, 121] |
| MTL Tauvid | 49 [42, 56] | 81 [73, 88] | 112 [102, 122] | 143 [129, 158] |
| Memory | 77 [68, 87] | 121 [108, 133] | 164 [146, 183] | 208 [182, 233] |
| Neocortex MK6240 | 94 [85, 103] | 129 [118, 140] | 163 [147, 179] | 198 [176, 219] |
| Executive function | 72 [60, 84] | 132 [114, 151] | 193 [164, 223] | 254 [212, 296] |
| Neocortex Tauvid | 127 [113, 142] | 171 [149, 192] | 214 [184, 244] | 257 [217, 297] |
| Attention | 118 [98, 138] | 186 [152, 220] | 254 [203, 305] | 322 [253, 390] |
| Language | 118 [97, 140] | 192 [154, 230] | 265 [207, 322] | 338 [261, 416] |
| Visuospatial | 174 [120, 228] | 274 [180, 368] | 373 [237, 510] | 473 [294, 652] |

CI: confidence interval; MTL: medial temporal lobe; prob: probability.

**Supplemental Figures**

## Supplemental Figure 1. Study flowchart.

Supplemental Figure 2. Sensitivity analysis for the discriminative accuracy of Tauvid and MK6240 tau PET for distinguishing clinical groups. Comparison of (A) MCI due to AD versus MCI due to other causes and (B) AD dementia versus MCI due to other causes. Participants with MCI due to AD or AD dementia were, by definition, Aβ-positive. Participants with MCI due to other causes were, by definition, Aβ-negative. Aβ: amyloid-β; AD: Alzheimer's disease; MCI: mild cognitive impairment.

Supplemental Figure 3. Diagnostic performance of Tauvid and MK6240 for differentiating clinical groups. Receiver operating characteristic (ROC) curves compare Tauvid and MK6240 in the medial temporal lobe for discriminating between cognitively impaired (CI) Aβ– and mild cognitive impairment (MCI) Aβ+ (left), and between CI Aβ– and Alzheimer’s disease (AD) dementia (right). Asterisks indicate significantly higher area under the curve (AUC) values for MK6240 compared with Tauvid (DeLong test, p < 0.05).

Supplemental Figure 4. Sensitivity analysis for the frequency of Tauvid and MK6240 tau positivity with the cognitively impaired group separated by diagnosis. Cumulative tau positivity in the medial temporal lobe (MTL; solid lines) and neocortex (Neo; dashed lines) of Tauvid (red) and MK6240 (blue) as a function of Aβ burden (Centiloid) in (A) mild cognitive impairment (MCI) and (B) dementia participants. P values indicate whether detection of tau positivity differs between MK6240 and Tauvid. Analyses adjusted for age, sex, and site. Aβ: amyloid-β; PR: prevalence ratio.

Supplemental Figure 5. Average SUVR images for Tauvid and MK6240 across Centiloid (CL) ranges. MK6240 demonstrated greater and more widespread binding than Tauvid at comparable Centiloid levels, with cortical involvement becoming extensive at higher Centiloid values. SUVR: standardised uptake value ratio.

Supplemental Figure 6. Tau tracer MTL and neocortical positivity stratified by Centiloid and clinical status. Tau positivity increased with levels of amyloid-β burden, with MK6240 consistently identifying more tau-positive individuals than Tauvid in the (A) medial temporal lobe and (B) neocortical region. CL: Centiloid.

Supplemental Figure 7. Comparison of Tauvid and MK6240 tau positivity rates in the medial temporal lobe. (A) Scatter plot showing the relationship between individual-level predicted probabilities of tau positivity for Tauvid and MK6240, derived from separate logistic regression models (tau positivity ~ Centiloid). Each point represents one participant from the full sample. The diagonal dashed line represents the line of identity (equal sensitivity). When MK6240 reaches 50% positivity, Tauvid is at 27.5%; when Tauvid reaches 50%, MK6240 is at 75%. The non-linear deviation from the identity line demonstrates MK6240's greater positivity at earlier disease stages. (B) The average rate of positivity increase per 10 Centiloid units, calculated from the derivative of logistic regression curves. Error bars represent 95% confidence intervals. MK6240 peaks earlier (50-60 Centiloids) than Tauvid (70-80 Centiloids), while memory impairment increases gradually.

Supplemental Figure 8. Cross-correlation and agreement between Tauvid and MK6240 in medial temporal lobe and neocortex. Scatter plots showing the relationship between Tauvid and MK6240 standardized uptake value ratios (SUVR) in the medial temporal lobe (A) and neocortical late-stage regions (B). Each point represents one participant, color-coded by cognitive status. Vertical and horizontal dashed lines indicate tracer-specific positivity thresholds. R² values demonstrate linear correlations between tracers in both regions. Bland-Altman plots showing the difference between MK6240 and Tauvid SUVR values (y-axis) against their average (x-axis) for all participants (C-D) and tau-positive individuals only (E-F). The solid horizontal line represents mean differences, dotted horizontal lines indicate 95% limits of agreement. The solid black regression line demonstrates proportional bias.

Supplemental Figure 9. Patterns of tau PET uptake in illustrative cases scanned with both Tauvid and MK6240. These cases highlight instances of tracer-specific differences in medial temporal lobe (MTL) binding within the same individuals. SUVR: standardised uptake value ratio. Aβ: amyloid-β; CU: cognitively unimpaired; MCI: mild cognitive impairment.

Supplemental Figure 10. Distribution of diagnostic groups across tau PET positivity patterns. Stacked bar plots show the proportion of participants classified as cognitively unimpaired (CU) or cognitively impaired (CI), and as amyloid-β negative (Aβ–) or amyloid-β positive (Aβ+), within each tau PET positivity group: both tracers negative, Tauvid-positive only, MK6240-positive only, and both tracers positive. Percentages are expressed relative to the total number of participants within each category.

Supplemental Figure 11. Sensitivity analyses using alternative MTL tau-positivity cut-off definition methods. Thresholds were defined as 2.0, 2.25, 2.5, 2.75, and 3.0 standard deviations (SD) above the mean of three control populations: (A) young adults (18-28 years old), (B) middle-aged cognitively unimpaired Aβ-negative adults (50-65 years old), and (C) cognitively unimpaired elderly Aβ-negative individuals (50-87 years old). (left) Number of individuals classified as Tauvid+ only, MK6240+ only, or both positive across each threshold. (right) Logistic regression curves relating Centiloid burden to tau-positivity for Tauvid and MK6240, with line thickness indicating the applied threshold. Insets show Centiloid estimates with 95% confidence intervals for the level associated with 50% positivity at each threshold.

Supplemental Figure 12. Sensitivity analysis of discordant classifications of tau positivity with Tauvid and MK6240 using quantitative criteria only. (A) Average standardised uptake value ratios (SUVR) maps of individuals positive only for Tauvid (top) and only for MK6240 (bottom). Distribution of (B) Aβ positivity, (C) APOEε4 carriership, (D) Centiloid levels and (E) memory composite score according to Tauvid and MK6240 status. Data are represented as median ± interquartile interval. Both negative: individuals negative on Tauvid and MK6240. Both positive: individual positive on Tauvid and MK6240.

Supplemental Figure 13. Regional positivity frequencies across brain regions comprising Braak stages for Tauvid and MK6240 in Aβ+ participants. The top panel shows the percentage of Aβ-positive participants classified as tau-positive in each cortical region for Tauvid and MK6240. The bottom panel depicts the difference in positivity frequency between tracers (MK6240 − Tauvid). MK6240 shows consistently higher positivity rates across all cortical regions.

Supplemental Figure 14. Relationship between amyloid-β burden and probability of tau positivity across Braak stages for Tauvid and MK6240. (A) Logistic models showing the probability of regional tau positivity as a function of Centiloid values for each Braak stage using Tauvid and MK6240. (B) Centiloid values corresponding to a 50% probability of positivity for each tracer and Braak stage. MK6240 curves and thresholds are systematically left-shifted relative to Tauvid, indicating that MK6240 detects tau pathology at lower amyloid-β burden levels.

Supplemental Figure 15. Concordance of Braak-like tau stages between Tauvid and MK6240. Mean (±SD) Braak stage for Tauvid given MK6240 stage (left) and for MK6240 given Tauvid stage (right). Across all stages, Tauvid consistently assigned lower Braak stages than MK6240, suggesting that MK6240 detects tau deposition at earlier or more advanced regional-involvement thresholds.

# References

1.	McKhann GM, Knopman DS, Chertkow H, et al. The diagnosis of dementia due to Alzheimer's disease: recommendations from the National Institute on Aging-Alzheimer's Association workgroups on diagnostic guidelines for Alzheimer's disease. *Alzheimer's & dementia : the journal of the Alzheimer's Association* 2011; **7**(3): 263-9.

2.	Kind AJH, Buckingham WR. Making Neighborhood-Disadvantage Metrics Accessible - The Neighborhood Atlas. *N Engl J Med* 2018; **378**(26): 2456-8.

3.	Dodge HH, Goldstein FC, Wakim NI, et al. Differentiating among stages of cognitive impairment in aging: Version 3 of the Uniform Data Set (UDS) neuropsychological test battery and MoCA index scores. *Alzheimers Dement (N Y)* 2020; **6**(1): e12103.

4.	Arani A, Borowski B, Felmlee J, et al. Design and validation of the ADNI MR protocol. *Alzheimers Dement* 2024; **20**(9): 6615-21.

5.	Fonov V, Evans AC, Botteron K, Almli CR, McKinstry RC, Collins DL. Unbiased average age-appropriate atlases for pediatric studies. *NeuroImage* 2011; **54**(1): 313-27.

6.	Klunk WE, Koeppe RA, Price JC, et al. The Centiloid Project: standardizing quantitative amyloid plaque estimation by PET. *Alzheimers Dement* 2015; **11**(1): 1-15.e1-4.

7.	Tward DJ, Sicat CS, Brown T, et al. Entorhinal and transentorhinal atrophy in mild cognitive impairment using longitudinal diffeomorphometry. *Alzheimers Dement (Amst)* 2017; **9**: 41-50.

8.	Kulason S, Tward DJ, Brown T, et al. Cortical thickness atrophy in the transentorhinal cortex in mild cognitive impairment. *Neuroimage Clin* 2019; **21**: 101617.

9.	Jack CR, Jr., Wiste HJ, Weigand SD, et al. Defining imaging biomarker cut points for brain aging and Alzheimer's disease. *Alzheimers Dement* 2017; **13**(3): 205-16.

10.	Pascoal TA, Therriault J, Benedet AL, et al. 18F-MK-6240 PET for early and late detection of neurofibrillary tangles. *Brain* 2020; **143**(9): 2818-30.
